# C-type lectin receptor expression is a hallmark of neutrophils infiltrating the skin in epidermolysis bullosa acquisita

**DOI:** 10.3389/fimmu.2023.1266359

**Published:** 2023-09-20

**Authors:** Christian F. Guerrero-Juarez, Paul Schilf, Jing Li, Maria Paula Zappia, Lei Bao, Payal M. Patel, Jenny Gieseler-Tillmann, Sripriya Murthy, Connor Cole, Maria Sverdlov, Maxim V. Frolov, Takashi Hashimoto, Norito Ishii, Thomas Rülicke, Katja Bieber, Ralf J. Ludwig, Christian D. Sadik, Kyle T. Amber

**Affiliations:** ^1^ Carle Illinois College of Medicine, University of Illinois, Urbana-Champaign, Urbana, IL, United States; ^2^ Department of Dermatology, Rush University Medical Center, Chicago, IL, United States; ^3^ Department of Dermatology, Allergy, and Venereology, University of Lübeck, Lübeck, Germany; ^4^ Department of Biochemistry and Molecular Genetics, University of Illinois at Chicago, Chicago, IL, United States; ^5^ Department of Dermatology, Massachusetts General Hospital, Boston, MA, United States; ^6^ Research Histology Core, University of Illinois at Chicago, Chicago, IL, United States; ^7^ Department of Dermatology, Osaka Metropolitan University Graduate School of Medicine, Osaka, Japan; ^8^ Department of Dermatology, Kurume University School of Medicine, and Kurume University Institute of Cutaneous Cell Biology, Kurume, Japan; ^9^ Department of Biomedical Sciences and Ludwig Boltzmann Institute for Hematology and Oncology, University of Veterinary Medicine Vienna, Vienna, Austria; ^10^ Lübeck Institute of Experimental Dermatology, University of Lübeck, Lübeck, Germany; ^11^ Department of Internal Medicine, Rush University Medical Center, Chicago, IL, United States

**Keywords:** epidermolysis bullosa acquisita, pemphigoid, neutrophil, single cell RNA seq, C-type lectin receptor (CLRs)

## Abstract

**Introduction:**

Inflammatory epidermolysis bullosa acquisita (EBA) is characterized by a neutrophilic response to anti-type VII collagen (COL7) antibodies resulting in the development of skin inflammation and blistering. The antibody transfer model of EBA closely mirrors this EBA phenotype.

**Methods:**

To better understand the changes induced in neutrophils upon recruitment from peripheral blood into lesional skin in EBA, we performed single-cell RNA-sequencing of whole blood and skin dissociate to capture minimally perturbed neutrophils and characterize their transcriptome.

**Results:**

Through this approach, we identified clear distinctions between circulating activated neutrophils and intradermal neutrophils. Most strikingly, the gene expression of multiple C-type lectin receptors, which have previously been reported to orchestrate host defense against fungi and select bacteria, were markedly dysregulated. After confirming the upregulation of *Clec4n*, *Clec4d*, and *Clec4e* in experimental EBA as well as in lesional skin from patients with inflammatory EBA, we performed functional studies in globally deficient *Clec4e^−/−^* and *Clec4d^−/−^* mice as well as in neutrophil-specific *Clec4n^−/−^* mice. Deficiency in these genes did not reduce disease in the EBA model.

**Discussion:**

Collectively, our results suggest that while the upregulation of *Clec4n*, *Clec4d*, and *Clec4e* is a hallmark of activated dermal neutrophil populations, their individual contribution to the pathogenesis of EBA is dispensable.

## Introduction

Epidermolysis bullosa acquisita (EBA) is a rare chronic skin condition caused by autoantibodies to type VII collagen (COL7). Within EBA, there are several distinct clinical phenotypes (1, 2). The inflammatory (bullous pemphigoid-like) phenotype is characterized by pruritis, dermatitis, and bullae. Histologically, inflammatory EBA is characterized by an abundance of neutrophils that release, among others, reactive oxygen species (ROS), leukotriene B_4_ (LTB_4_), and proteases at the cutaneous basement membrane zone, resulting in skin blistering (3–7). Neutrophils are the most abundant leukocyte in human blood. Circulating neutrophils demonstrate limited antimicrobial activity (8). However, after priming with various molecules, such as proinflammatory cytokines and chemokines, as well as microbial products, neutrophils gain improved phagocytic capacity and ROS production, and demonstrate a distinct surface phenotype (9, 10). CD54, dectin-2, and IL-1β expression is acquired by neutrophils during the late phase of priming as they prepare for migration to inflammatory sites (11). Dectin-2 (CLEC6A/CLEC4N) belongs to the C-type lectin-like family of receptors (CTLR) (12), a family of transmembrane pattern recognition receptors expressed on myeloid cells. These CTLRs recognize pathogen-associated molecular patterns as well as certain modified self-antigens, such as damage-associated molecular patterns released from dead cells.

While neutrophil priming was previously thought to occur in the absence of transcription or translation, several studies have refuted this notion (11). Recent technological advances have allowed detection of neutrophil transcriptomes in spite of their low RNA content, revealing their heterogeneity in the blood and in host defense (13). Yet, the distinction between skin-infiltrating versus blood neutrophils remains largely unknown. While several phenotypic neutrophil activation markers have been identified, it is likely that these skin-infiltrating neutrophils have a distinct phenotype from blood and even activated blood neutrophils, as has been shown in other tissues (14).

As neutrophils play a central role in innate immunity, the concept of global therapeutic inhibition of neutrophils would be associated with significant potential side effects. However, targeting markers present only on neutrophils contributing towards disease pathology would potentially bypass this risk. Aside from identifying targetable surface markers on neutrophils, identification of intradermal function is of great importance in disease, as they may contribute to local inflammation. In psoriasis, for example, neutrophils are a source of IL-17 (15).

The identification of novel disease-specific neutrophil markers is complicated, among others, by the fact that neutrophils are sensitive cells, susceptible to both swift activation and apoptosis in response to manipulation. To identify characteristic markers of cutaneous neutrophils in EBA, we performed single-cell RNA-sequencing (scRNA-seq) on unsorted blood and skin from mice in the antibody transfer model of EBA. By minimizing enrichment steps and manipulation time, this approach permitted us to capture viable neutrophils, allowing bioinformatic analysis of neutrophil activation from blood to skin.

## Materials and methods

### Animal experiments

Animal experiments were approved by the Animal Care and Use Committee at the Rush University Medical Center (IACUC No 20-079) or the state government of Schleswig-Holstein (Protocol 119-10/17), depending on where the experiments were conducted. All experiments were performed by certified personnel.

### Human samples and ethics statement

Tissue from five patients with confirmed inflammatory EBA, defined as a neutrophilic inflammatory infiltrate, positive direct immunofluorescence, and positive dermal side indirect immunofluorescence with corresponding anti-COL7 IgG, was retrospectively collected. All patients involved in this study provided written informed consent. All experiments with human samples were approved by the ethical committee of the Rush University Medical Center (IRB No. 20121406) and were performed in accordance with the Declaration of Helsinki.

### Mice

C57BL6/J were purchased from Jackson Laboratories (Bar Harbor, ME, USA) (JAX stock 000664). Sperm of C57BL/6*-Clec4d^tm1.1Cfg/Mmucd^* (*Clec4d^−/−^*; MMRRC stock: 031935-UCD) and C57BL/6*-Clec4e^tm1.1Cfg/Mmucd^* (*Clec4e^−/−^*) mice (MMRRC stock 031936-UCD), designated with the MMRCR stock numbers 031935-UCD and 031936-UCD, respectively, were purchased from the Mutant Mouse Research and Resource Center (MMRRC; Davis, CA, USA). The sperm was used for *in vitro* fertilization. The heterozygous progeny was used to breed *Clec4d^−/−^* and *Clec4e^−/−^* mice and their respective wild-type littermates. *MRP8-Cre-ires/GFP* (MRP8-Cre) mice were obtained from the Jackson Laboratory (Bar Harbor, ME, USA). A conditional knockout of *Clec4n* was generated using *Clec4n^tm2a(KOMP)Wtsi^* embryonic stem (ES) cells acquired from the KOMP Repository (Davis, CA, USA). Using the fully verified and karyotyped C57BL/6N ES cells, chimeric founder mice were generated by microinjection into BALB/c blastocysts. The resulting mice with the “knockout first allele” were crossed with a C57BL/6N-Tg(CAG-Flpe) deleter mouse to remove the selection cassette and create the conditional *Clec4n^tm1c^* allele, hereafter referred to as *Clec4n^fl^* allele. Polymorphonuclear neutrophil (PMN)-specific *Clec4n^−/−^* (*Clec4n^ΔPMN^*) mice were generated by crossing homozygous *Clec4n^fl/fl^* with MRP8-Cre mice inducing an MRP8 promotor-driven Cre recombination excision event during neutrophil differentiation of precursors. To confirm the gene knockout in neutrophils, bone marrow neutrophils were isolated using the Neutrophil Isolation Kit (Miltenyi, Teterow, Germany), and their RNA was isolated using the RNA Mini Kit (Analytik Jena AG, Jena, Germany). cDNA was generated using the Thermo Scientific Revert Aid First Strand cDNA Synthesis Kit (Thermo Fisher Scientific, Bremen, Germany). cDNA was used for quantitative PCR using the SYBR™ Select Mastermix (Thermo Fisher Scientific, Bremen, Germany). Data were acquired using the RealPlex (Eppendorf, Hamburg, Germany) cycler (data not shown).

### Generation of COL7 ^vWFA2^

Recombinant murine von Willebrand factor A-like domain 2 (vWFA2) of the NC1 domain of collagen 7 (aa1048-1238) was produced, as previously described (16, 17).

### Development of anti-murine COL7^vWFA2^ IgG

New Zealand white rabbits were immunized with COL7^vWFA2^ as previously described (18). IgG was purified from rabbit serum using Protein G Sepharose Fast Flow affinity column chromatography (Amersham Biosciences, Freiburg, Germany) as previously described (18). Reactivity of IgG fractions was analyzed by the immunofluorescence microscopy on murine skin. Concentrations of purified rabbit IgG were measured at 280 nm by a spectrophotometer. Total rabbit IgG underwent an additional purification step of antigen affinity purification using COL7^vWFA2^ coupled Affi-Gel 10 (Bio-Rad, Munich, Germany) as previously described (19).

### Induction of experimental EBA

Mice were housed under specific pathogen-free conditions at Rush University (Chicago, IL, USA) or at the University of Lübeck (Lübeck, Schleswig-Holstein, Germany) and were provided standard mouse chow and acidified drinking water *ad libitum*. Mice were conditioned at least 2 weeks prior to experiments and were under 12 h:12 h light/dark cycles. Sex-matched mice, age 6–12 weeks were used for the experiments. For transcriptomic analyses, antibody transfer EBA was performed, as previously described (17). Affinity-purified anti-COL7^vWFA2^ IgG (200 µg) was injected intraperitoneally thrice weekly for 2 weeks. In experiments examining the contribution of Dectin-2, Dectin-3, and Mincle to experimental EBA, disease was induced and evaluated, as previously described (4, 20). Briefly, New Zealand rabbits were immunized against three epitopes of type VII collagen. IgG directed to the epitope C (anti-COL7c) was affinity purified, and 50 μg of anti-COL7c was injected subcutaneously on days 0, 2, and 4 of the experiment. The percentage of the total body surface area affected by skin lesions (erythema, blisters, erosions, crusts, or alopecia) was determined by an investigator blinded to the experimental conditions. All clinical examinations and bleedings were performed under anesthesia.

### Histology and immunofluorescence

For mouse tissue, additional perilesional tissue from previous passive transfer experiments and optimizations were used to minimize animal numbers. Hematoxylin and eosin stains were performed using standard protocols. For both human and mouse, FFPE blocks were sectioned routinely and stained with the following antibodies: CLE4D (MBS9607710, rabbit polyclonal, Mybiosource, San Diego, CA, USA), CLEC4E (BS08541R, rabbit polyclonal, Bioss, Woburn, Massachusetts, USA), and CLEC4N (clone IMG3D1, ab107572, mouse IgG3 monoclonal, Abcam, Boston, Massachusetts, USA). Human neutrophils were stained for MPO (AF3667, goat polyclonal, R&D Systems, Minneapolis, Minnesota, USA), while mouse samples were stained for Ly6G (clone RB6-8C5, SC-53515, rat monoclonal IgG2b, Santa Cruz, Dallas, Texas). Secondary antibodies and DAPI counterstain were purchased from ThermoFisher (Rockford, IL, USA). Mouse direct immunofluorescence was performed as previously described, using anti-rabbit secondary antibody to confirm successful passive transfer (17). All slides were immediately photographed following staining using an Evos FL microscope (ThermoFisher, Rockford, IL, USA). The frequency of dual-positive cells was quantified in ImageJ v1.52 (Bethesda, Maryland, USA)

### Flow cytometry

Surface antigen staining was performed according to standard flow cytometry procedures. The following antibodies and reagents were used: Zombie aqua viability stain, anti-Ly6G (clone 1A8, 127628, Rat IgG2a), anti-CD45 (clone 30-F11, 103151, Rat IgG2b), and anti-CD11b (clone M1/70, 101243, Rat IgG2b), all of which were purchased from BioLegend (San Diego, CA, USA). Anti-CLEC4D (clone MA5-24152, mouse IgG2b) was purchased from ThermoFisher (Rockford, IL, USA) and conjugated with APC using the Lightning-Link APC Conjugation Kit (Abcam, Boston, Massachusetts, USA). Recombinant anti-CLEC4N targeting the extracellular domain (50267-R001, Rabbit IgG, Sino Biological, Wayne, PA, USA), was conjugated with PE using the Lightning-Link PE Conjugation Kit (Abcam, Boston, Massachusetts, USA) and anti-CLEC4E (BS-8541R, Rabbit polyclonal, ThermoFisher, Rockford, IL, USA), was conjugated with Alexafluor-488 using the Lightning-Link Alexafluor488 Conjugation Kit (Abcam, Boston, Massachusetts, USA). Flow cytometry was performed on an LSRFortessa (BD Biosciences, San Jose, CA, USA), using compensation beads (BioLegend, San Diego, CA, USA) and FACSDiva software (BD Biosciences, San Jose, CA, USA). Gating was performed by removing doublets and dead cells, followed by use of fluorescent minus one control. Flow cytometry data were analyzed using FCS Express 7 Plus software (*De Novo* Software, Pasadena, CA, USA).

### Tissue harvesting for single-cell RNA-sequencing

Blood was collected by retrobulbar bleeding from anesthetized mice, and immediately followed by RBC lysis (BioLegend, San Diego, CA, USA). Lesional (ear) skin from anesthetized mice was harvested by punch biopsies and enzymatically digested using the whole skin digestion kit (Miltenyi, Auburn, CA, USA) per manufacturer instructions with minor modifications. Following addition of enzymes, samples were incubated in a water bath at 37°C for 2 h and shaken every 15 min. Mechanical dissociation by pipetting was only performed at the end of the incubation. Cells were then filtered using a 70-μm filter, centrifuged at 300*g* for 10 min at 4°C, washed and resuspended in 0.04% BSA/HBSS (ThermoFisher, Rockford, IL, USA). Cell viability was immediately assessed by trypan blue (ThermoFisher, Rockford, IL, USA). Single-cell suspensions of 1,000 cells/μL were run into the chromium Controller (10x Genomics, Pleasanton, CA, USA) to encapsulate 5,000 and 10,000 cells for blood and skin samples, respectively. Ambion RNase inhibitor (Invitrogen, Waltham, Massachusetts, USA) was added to the master mix. Samples were processed using the Chromium Single Cell 3′ GEM, Library & Gel Bead Kit v3 (10x genomics, Pleasanton, CA, USA) per manufacturer’s instructions. Library QC was performed using the Agilent Tape station. Sequencing was performed using two NovaSeq 6000 SP lanes (Illumina, San Diego, CA, USA). Cell capture, RT, library preparation, and sequencing were performed at the University of Illinois at Chicago.

### Single-cell RNA-sequencing and bioinformatics

Raw FASTQ reads were mapped to mouse mm10 reference genome (GRCm38.93.dna/GRCm38.93.gtf) using Cell Ranger Version 3.0.0. Web summary alignment metrics are provided in **Supplementary Table 3**.

### Doublet/multiplet simulation and low-quality cell pruning

Raw, digitized count matrices were pre-processed and doublets/multiplets were simulated using Single-Cell Remover of Doublets (Scrublet) (21) (version 0.2.1) with standard parameters enabled. Putative singlets were filtered in Seurat to remove low-quality cells and kept for downstream analysis if and only if they met the following user-defined, collective quality control metrics criteria: (a) 350 < genes/cell < 5,000, (b) cells contained no more than 10% mitochondrial gene expression, and (c) cells were not defined as outliers (22).

### Anchoring, integration, and downstream analysis

We performed anchoring and integration of pathogenic IgG or non-pathogenic IgG-treated mouse blood or pathogenic IgG or non-pathogenic IgG-treated mouse skin datasets using the Seurat package (Version 3.2.2, R Studio version 3.6.1) (23) as suggested by the developer. Briefly, Seurat objects were created using Scrublet-pre-processed individual, raw digitized count matrices and merged. Individual gene expression digital matrices were normalized, and the top 2,000 variable genes/features were identified. Integration anchors were identified using the dimensions set to 30, and datasets were integrated with dimensions set to 30. The integrated object was scaled, and significant principal components used for clustering and finding neighbors were identified using a combination of statistical and heuristic methods. Neighbors and clusters were then identified with dimensions specified by user and visualized using Uniform Manifold Approximation and Projection (UMAP). Putative cell identities were identified based on differential gene expression profiles (cluster markers) (log fold change of 25% with a minimum of 25% of cells expressing such gene in either one cluster). Genes were log-normalized and visualized as two-dimensional feature plots or bubble plots.

### Differential gene expression analysis

Differentially expressed genes between cell types across treatments were calculated with Seurat. Differentially expressed genes for a particular comparison were filtered using a *p*-adjusted threshold of 0.05 and a Log_2_ fold-change of 0.58 or greater in either direction.

### Gene class and GO analysis

Differentially expressed genes were used to calculate Gene Ontologies (GOs). Manual curation of GOs was performed using Enrichr (24) and visualized as bubble plots. A list of significant GOs (*p*-adjusted < 0.05) is included in **Supplementary Table 4**.

### Marker gene module scoring

Aggregate, composite gene scores were assigned to neutrophils using the *AddModuleScore* function in Seurat. This *“Neutrophil aggregate score”* was defined by a core set of known neutrophil-associated marker genes, including *S100a8*, *S100a9*, and *Ly6g*. Aggregate, composite gene scores were log-normalized and visualized as two-dimensional feature plots.

### Pseudotime analysis

Pseudo-ordering of individual cells was performed using Monocle2 (Version 2.10.1) (25). Briefly, neutrophils were bioinformatically gated in Seurat and a *cellDataSet* object was created in Monocle2. Subclustered cells were ordered based on variable features and dimensionality reduction was performed with the *reduction method* = *’DDRTree’* argument enabled and then ordered. To identify pseudotime-dependent gene expression changes in neutrophils in the putative blood–skin transition, we applied the single-cell Energy path (scEpath; Version 1; MATLAB Version 9.5) (26) algorithm on a subset of Monocle2-ordered neutrophils. To identify statistically significant pseudotime-dependent gene changes, we compared the standard deviation of the observed smoothed expressions with a set of similarly permuted expressions by randomly permuting the cell order (i.e., 100 permutations) as suggested by the developer. We considered all genes with a standard deviation greater than 0.01 and a Bonferroni-corrected *p-*value below a significance level *α* = 0.05 to be pseudotime-dependent. To analyze pseudotime-dependent mouse transcription factors, we used genes annotated in the Animal Transcription Factor Database (AnimalTFDB 3.0) (27) in scEpath. Pseudotime-dependent genes were represented and visualized using a rolling wave plot with user-defined optimal *K* clusters.

### Statistical analyses

Percentages of CLEC4-positive neutrophils between blood in EBA versus control or EBA blood versus skin were determined by log-transformation of the percentage of dually positive cells, followed by Student’s *t*-test. Comparisons of the body surface area affected by skin lesions were performed by two-way ANOVA using GraphPad Prism 9 (GraphPad, San Diego, CA, USA), and comparisons of the area under the curve of the affected body surface area over time were performed using Student’s *t*-test. All tests were two sided with a *p* < 0.05. Given the relatively few numbers of comparisons and lack of statistically significant results, correction for multiplicity was not performed. All data are reported as mean ± SEM.

## Results

### A single-cell repertoire of whole blood and lesional skin in experimental EBA

**Figure 1:**
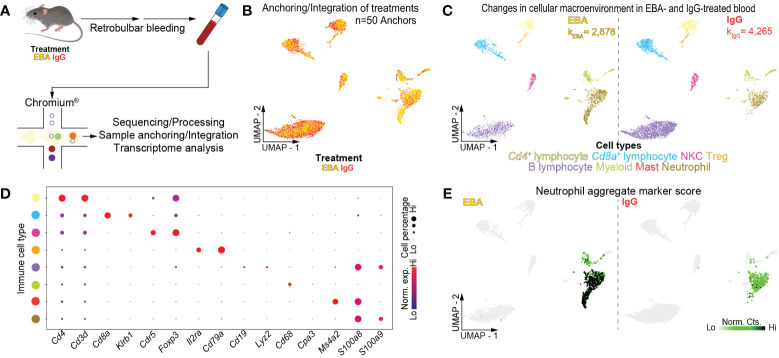
Single-cell transcriptome of mouse blood in experimental EBA. **(A)** Schematic of blood cell isolation, processing, and capture by droplet-based device, 3′-scRNA-seq, and downstream analyses. **(B)** Anchoring of EBA or control mouse leukocytes into a single object and visualized in two-dimensional UMAP space. **(C)** Clustering and neighbor identification of anchored datasets. Eight putative immune cell populations were identified and color coded. Putative cluster identity based on *bona fide* marker gene expression is defined on the bottom. **(D)** Dot plot of key markers of each immune population with **Figure 1C**. **(E)** Two-dimensional feature plot showing expression of aggregate, composite neutrophil gene score. Light green—low, black—high gene expression based on normalized counts. Data shown are from *n* = 3 pooled biologic replicates per condition. EBA, epidermolysis bullosa acquisita; IgG, immunoglobulin G; UMAP, Uniform Manifold Approximation and Projection; NKC, natural killer cell; Treg, regulatory T cell; Norm. exp., normalized expression; Lo, low; Hi, high; Norm, Cts, normalized counts.

**Figure 2:**
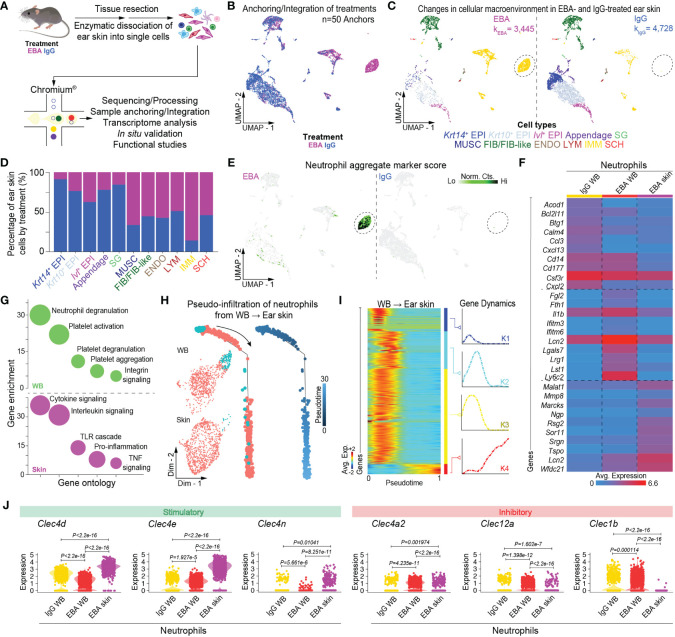
Single-cell transcriptome profiling of skin lesions in experimental EBA. **(A)** Schematic of ear skin cell isolation workflow. **(B)** Anchoring of skin cells from EBA or control mice into a single object and visualized in two-dimensional UMAP space. **(C)** Clustering and neighbor identification of anchored datasets. Eleven putative cell populations were identified and color coded. Putative cluster identity based on *bona fide* marker gene expression is defined on the bottom. Major cellular identities of recovered cells reveal a unique population of immune cells in the EBA relative to control group (circled). **(D)** Relative frequency of putative cellular identities of each cluster in EBA (magenta) and IgG control groups (blue). Cells from the EBA group contributed to a far greater number of immune cells, while a greater frequency of keratinocytes were recovered from the control group as expected. **(E)** Two-dimensional feature plot showing expression of aggregate, composite neutrophil gene score demonstrates the immune population unique to EBA skin to be neutrophils. Light green—low, black—high gene expression based on normalized counts. Data shown are from *n* = 3 pooled biologic replicates per condition. **(F)** Heatmap of differentially expressed genes between neutrophils from the skin and whole blood (LogF.C. = 0.25×, 25% cell/cluster, Wilcoxon Rank Sum Test *p* < 0.05). The top 10 upregulated genes from the following three comparisons are shown: control blood neutrophils versus EBA blood neutrophils; EBA blood neutrophils versus control blood neutrophils; and EBA skin neutrophils versus all blood neutrophils (control and EBA). **(G)** Gene ontology for biological process of differentially expressed genes (LogF.C. = 0.25×, 25% cell/cluster, Wilcoxon Rank Sum Test *p* < 0.05) in the cutaneous and activated neutrophil population respectively versus basal neutrophils. **(H)** Trajectory analysis in pseudotime depicting a pseudo-infiltration of neutrophils from blood into ear skin. **(I)** Rolling wave plot of pseudotemporal total gene (*k*=4) expression. Pseudotemporal gene expression is based on normalized counts. Blue—downregulated genes; Red—upregulated genes. **(J)** Violin plots demonstrate a relative increase in stimulator CTLR gene expression and decrease in inhibitory CTRL in EBA skin relative to blood neutrophils. EBA, epidermolysis bullosa acquisita; IgG, immunoglobulin G; UMAP, Uniform Manifold Approximation and Projection; EPI, epithelium; SG, sebaceous gland; MUSC, muscle; FIB/FIB-like, fibroblast/fibroblast-like; ENDO, endothelial; LYM, lymphatic; IMM, immune; SCH, Schwann; Norm. exp., normalized expression; WB, whole blood; Avg. Exp., average expression; Lo, low; Hi, high; Norm, Cts, normalized counts.

Mice were injected thrice weekly for 2 weeks with pathogenic rabbit anti-mouse antigen affinity-purified COL7^vWFA2^ IgG or with non-pathogenic rabbit IgG, as previously described (17). As expected, the group treated with pathogenic IgG consequently developed a characteristic disease phenotype with positive direct immunofluorescence against the basement membrane zone. We then performed scRNA-seq on unsorted cells from RBC-lysed blood (**Figure 1A**) or ear skin (**Figure 2A**) in experimental EBA mice or controls at day 14. Following doublet removal and quality control filtering (**Supplementary Figure 1**), 7,143 cells (k_EBA_ = 2,878 vs. k_IgG_ = 4,265) were anchored and integrated and included in our blood analysis (**Figures 1B, C**), and 8,173 cells (k_EBA_ = 3,445 vs. k_IgG_ = 4,728) in our skin analysis (**Figures 2B, C**).

### Assessment of immune cell heterogeneity in experimental EBA reveals a signature-activated neutrophil transcriptome

Unsupervised clustering of blood cells was performed with Seurat (28), revealing eight distinct clusters. Using differentially expressed gene signatures, we assigned clusters based on their putative identities and hierarchical similarities. As expected, these clusters were primarily composed of immune cells (**Figures 1C, D**), of which lymphocytes were most abundant followed by neutrophils (**Supplementary Figure 2**). We next compared the ratio of immune cells per cluster post-normalization between the pathogenic IgG and the non-pathogenic IgG-treated groups to identify shifts in immune populations because of disease. Neutrophils and myeloid cells appeared in larger amounts in samples from pathogenic IgG-treated mice relative to non-pathogenic IgG-treated mice (**Supplementary Figure 2**). Putative neutrophils were identified based on an aggregate, composite gene score including *S100a8*, *S100a9*, and *Ly6g*. Notably, two-dimensional UMAP demonstrates an apparent neutrophil activation in EBA samples that is absent in IgG control samples, seen in an expanding population emanating from the basal neutrophil populations (**Figure 1E**). Bioinformatic gating was subsequently performed to isolate neutrophils based on these putative markers, with resolution decreased to consider neutrophils as a single, granular population for each treatment condition. Wilcoxon Rank Sum Test revealed 148 upregulated and 375 downregulated genes between neutrophils from the pathogenic IgG and the non-pathogenic IgG-treated groups, defined as >0.25 fold-change difference, and a *p*-adjusted < 0.05 (**Supplementary Table 1**).

### Assessment of cellular heterogeneity in experimental EBA skin identifies the transcriptome of a cutaneous neutrophil population

We next characterized the cutaneous transcriptome in active lesions, with an emphasis on capturing viable lesional-skin neutrophils. Given neutrophil fragility following skin dissociation and flow sorting, dead cell depletion, or alternative dissociation protocols (unpublished), we capitalized on our scRNA-seq workflow to minimize such manipulation. Thus, following dissociation, whole skin dissociates were immediately processed for single-cell capture. Viability threshold of >80% was set without dead cell depletion to minimize dead cell background signal but allow capture of viable neutrophils. Using differentially expressed gene signatures, we assigned clusters based on their putative identities and hierarchical similarities as before (**Figure 2D**, **Supplementary Table 2**). Use of our putative neutrophil aggregate marker score highlighted a cutaneous neutrophil population in diseased but not IgG control skin (**Figure 2E**).

### Trajectory analysis reveals activation of neutrophils from blood to lesional skin

To better understand the trajectory of neutrophil activation, whole blood and skin neutrophils were combined into a single object using bioinformatic gating. Cells were then aligned in pseudotime using Monocle2 (25), with pseudotime-dependent genes identified in scEpath (26). Trajectory analysis revealed pseudo-infiltration from activated blood to intralesional neutrophils (**Figures 2H, I**). Control blood, EBA blood, and EBA skin each exhibited unique transcriptomes, of which the top 10 differentially expressed genes between each comparison are shown (**Figure 2F**). Gene ontology analyses with Enrichr (24) of the activated and cutaneous neutrophils versus basal neutrophils revealed increased neutrophil degranulation in blood and enhanced cytokine signaling in the skin (**Figure 2G**). While several genes demonstrated pseudotemporal activation from blood to skin, genes for CTRLs collectively displayed a clear pattern of regulation with several stimulatory CTLRs upregulated and inhibitory CTLRs downregulated (**Figure 2J**; **Supplementary Figure 3**). This intriguing finding prompted us to investigate these markers and their functional significance in more detail.

### Validation of Dectin-2, Dectin-3, and Mincle expression on neutrophils

**Figure 3:**
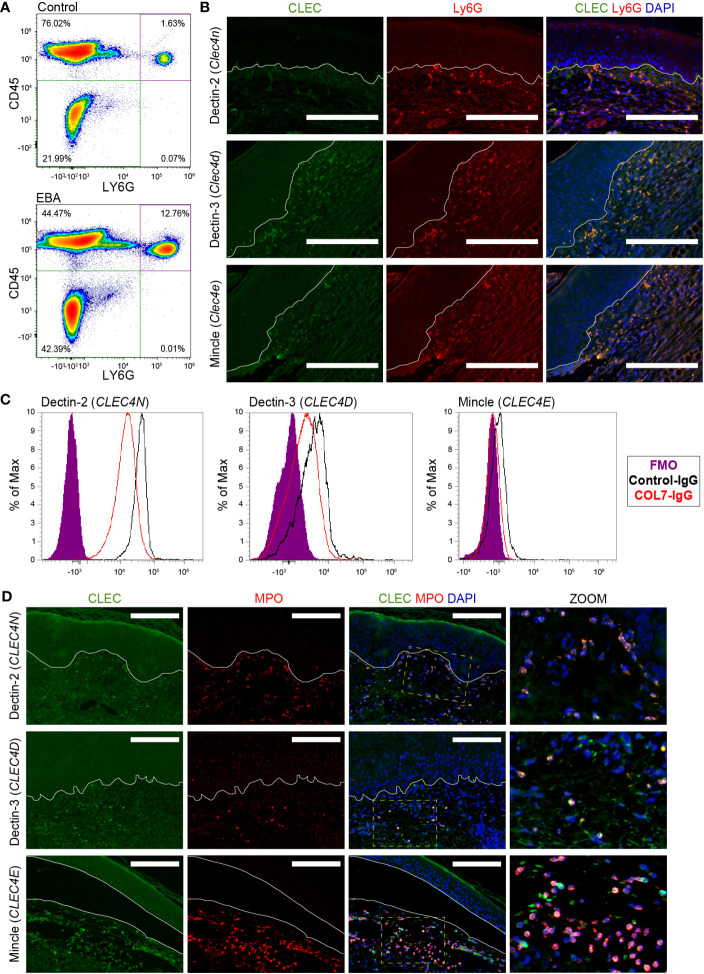
Expression of CLEC4 markers in human and experimental EBA neutrophils. **(A)** Flow cytometry of blood demonstrates an increased percentage of Ly6G^+^CD45^+^ cells in EBA relative to control mice. **(B)** Immunofluorescent staining of FFPE from mice with EBA reveals co-expression of Ly6G (green) and CLEC4 markers (red) overlaid with DAPI. Dectin-2, Dectin-3, and Mincle are expressed on a majority of neutrophils in experimental EBA. **(C)** Flow cytometry of blood neutrophils demonstrates that CLEC4N and CLEC4D are expressed on both control and EBA neutrophils, while CLEC4E is expressed only on a small number of neutrophils in both EBA and control mice (*n* = 3–4). **(D)** Immunofluorescent staining of FFPE from patients with inflammatory EBA demonstrates expression of CLEC4N/D/E (green) on MPO+ cells (red). Notes: Epidermis and dermis are demarcated by a white line. The inset demonstrates characteristic nuclear morphology of neutrophils on dually stained cells. Images from mouse tissue are representative of *n* = 10 from three independent experiments. Flow cytometry density plots are shown of a single biologic replicate and are representative of *n* = 3–4 mice. Images shown from patients with inflammatory EBA are representative of *n* = 5. Scale bars, 300 μm **(B)**; 300 μm **(D)**. EBA, epidermolysis bullosa acquisita; IgG, immunoglobulin G; FMO, fluorescent minus one.

Next, we characterized the protein expression levels of Dectin-3, Mincle, and Dectin-2, the proteins encoded by *Clec4d*, *Clec4e*, and *Clec4n*, respectively, on neutrophils. To this end, skin sections from mice with EBA were stained for Ly6G and either Dectin-3, Mincle, or Dectin-2 revealing co-expression in the skin (**Figure 3B**). To distinguish whether this activation occurred in the blood or in the skin, we performed flow cytometry on blood from mice treated with COL7 antibodies or corresponding isotype controls. As previously described (17), the frequency of CD45^+^Ly6G^+^ neutrophils increased significantly in the EBA compared to the control group (**Figure 3A**). Dectin-2 was highly expressed in both basal and activated blood neutrophils (>95%), while much lower levels of Dectin-3 and Mincle were detected. There was no significant difference between control and EBA neutrophils (**Figure 3C**) The percentage of neutrophils expressing Mincle and Dectin-3 was significantly increased in the skin relative to blood neutrophils (*p* < 0.001). We next sought to confirm the expression of these three CLTRs in human EBA patients by assessing formalin-fixed paraffin-embedded (FFPE) sections retrospectively. These sections were co-stained for myeloperoxidase (MPO) and either Dectin-2, Dectin-3, or Mincle. Skin neutrophils from patients with inflammatory EBA demonstrated consistent co-expression of Dectin-2, Dectin-3, and Mincle (**Figure 3D**). MPO-positive cells were uniformly positive for each CTLR. CTLRs were also detectable in few MPO-negative cells with a mononuclear morphology.

### Genetic deficiency in *Clec4d* and *Clec4e* and neutrophil-specific deficiency in *Clec4n* do not alter the course of EBA

**Figure 4:**
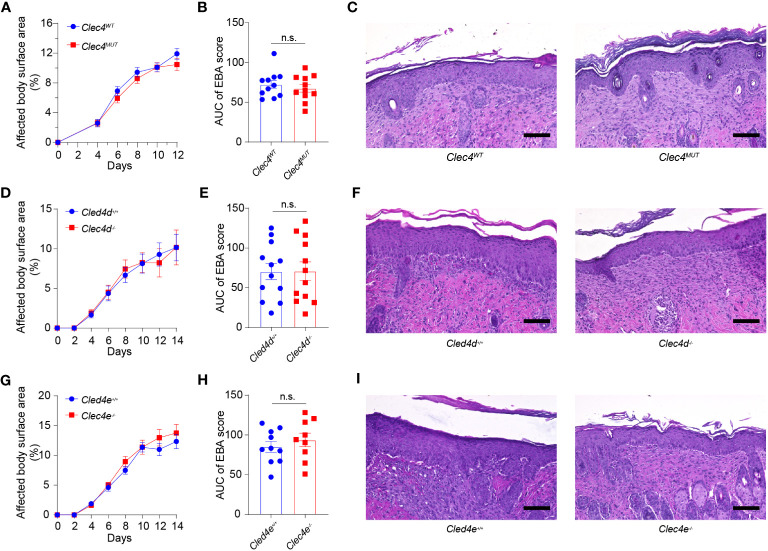
Genetic deficiency in *Clec4d*, *Clec4e*, and *Clec4n* in experimental EBA. **(A)** Affected Body Surface Area (BSA; clinical score) in antibody transfer-induced EBA in neutrophil-specific *Clec4n* (*Clec4n^ΔPMN^*) mutant mice). **(B)** Area under the curve analysis (AUC) of BSA over time. **(C)** Representative histology of CLEC4^MUT^ and CLEC4^WT^ following 2 weeks of antibody transfer reveals comparable inflammatory infiltrate. Similar findings are noted in the **(D)** BSA, **(E)** AUC, and **(F)** histology of *Clec4d^−/−^* vs. *Clec4e^+/+^* WT, as well as **(G)** BSA **(H)** AUC, and **(I)** histology of *Clec4e^−/−^* vs. *Clec4e^+/+^* WT. All results are presented as mean ± SEM. Data were merged from two to three independent experiments (*n* = 10–11 mice/group). **(A, D, G)** were analyzed by two-way ANOVA; **(B, E, H)** were analyzed by Student’s *t*-test. EBA, epidermolysis bullosa acquisita; BSA, body surface area; IgG, immunoglobulin G; AUC, area under the curve; n.s., not significant.

We analyzed the functional significance of Dectin-3, Mincle, and Dectin-2 in EBA. To this end, we examined the course of EBA in globally genetically deficient *Clec4d^−/−^* and *Clec4e^−/−^* mice as well as in neutrophil-specific *Clec4n^−/−^* mice. The latter were generated by breeding *Clec4n^fl/fl^* with *Mrp8^+/Cre^* mice. The clinical course of skin inflammation as well as the histopathology of skin lesions were analyzed, as described in the *Materials and methods* section. Genetic deficiency of each CTLR did not change the course of disease at the clinical or the histopathological level (**Figure 4**).

## Discussion

In recent years, evidence has accumulated that neutrophils are plastic cells phenotypically adapting to inflammatory conditions much more than previously anticipated and thus, at least temporarily, becoming a more heterogenous cell population. Understanding the plasticity and heterogeneity of neutrophils under inflammatory conditions is presumably key to elucidate the role of neutrophils in the pathogenesis of disease. Furthermore, they may offer the opportunity for therapeutic interventions specifically targeting those neutrophil subpopulations and functions driving disease.

Our knowledge of neutrophil heterogeneity is, however, still scarce, and predominantly generated from models of infectious diseases instead of sterile chronic inflammatory diseases. The latter, which include autoimmune diseases, are still mostly treated with immunosuppressive strategies associated with significant adverse events, particularly a high susceptibility to infections. They may therefore benefit the most from novel approaches specifically targeting pathogenic neutrophil subpopulations. The development of such approaches requires in-depth knowledge of neutrophil phenotypes under specific disease conditions. Our study reveals that in EBA, as a prototypical example of an organ-specific autoimmune disease affecting the skin, neutrophils significantly change their phenotype both at the transcriptional and protein level. These changes proceed both in the peripheral blood and in the skin, suggesting that both systemic and local signals initiate these shifts in the transcriptome.

Importantly, these findings lay the path to specifically target neutrophils responding to molecular signals derived from site of emerging peripheral sterile tissue inflammation. These strategies may, e.g., either inhibit select effector or regulatory functions or exploit cell surface markers unique among neutrophils for the pathogenic subpopulations for targeted cell depletion. In addition, the changes in the transcriptome of neutrophils in the peripheral blood may be of interest for the development of new biomarkers for the early detection of emerging tissue inflammation in autoimmune and other sterile inflammatory diseases.

A most striking difference between neutrophils in the peripheral blood and in lesional skin was the induction of the three stimulatory CTLRs, i.e., Dectin-2, Dectin-3, and Mincle. All three receptors were also expressed on neutrophils in lesional skin from EBA patients, suggesting that our mouse model faithfully reflects an aspect of the human situation. Collectively, these findings highlight these CTLRs as markers for activated neutrophils in lesional skin in EBA. They are in line and extend the findings of Yao et al. (11), which uncovered that Dectin-2 is upregulated on murine neutrophils upon migration into peripheral tissues. CTLRs play a critical role in host defense against fungal and bacterial pathogens. Dectin-2, for example, regulates Th17 responses to histoplasmosis and coccidioidomycosis and plays a protective role in streptococcal immunity (29–31). Dectin-2 additionally regulates ROS production and NADPH oxidase-independent NETosis in *Candida* infection (32–34). Mincle has a critical function in mycobacterial immunity (35). Dectin-2 and Mincle represent pattern recognition receptors, binding to polysaccharide moieties and resulting in activation of CARD9 and the Myd88 signaling cascade (36, 37). Dectin-3 has additionally a crucial role in mycobacterial immunity as well as in Gram-negative lung infections (38, 39). More recently, CTLRs have also been implicated in the pathogenesis of certain types of sterile tissue inflammation. For example, blockade of Mincle and Dectin-1 binding decreases murine neutrophil cytotoxicity towards tumor cells by inhibiting binding of CTLRs with nidogen-1 (40). Inhibition of Dectin-2 additionally confers resistance to house dust mite airway inflammation with decreased neutrophil influx (41). Dectin-2 can also regulate Th2 immunity through the generation of cysteinyl leukotrienes (42).

In our EBA mouse model, Dectin-2, Dectin-3, and Mincle were upregulated on neutrophils but genetic deficiency in mice for one or the other receptor did not alter the course of skin inflammation. This finding indicates that none of the receptors play a critical, non-redundant role in the regulation of neutrophil activities in EBA. However, as all three receptors were upregulated, we still cannot exclude that they might play a significant redundant role in this process. While neutrophil activation in experimental EBA occurs in a Syk-dependent manner (43), activation appears to occur through a different pathway than CTLR coupling with Syk kinase (44). The upregulation of multiple CTLR likely represents a conserved host response initiated by molecular signals shared by both microbial and sterile tissue inflammation rather than a disease-specific neutrophil phenotype. It is plausible that neutrophils recruited to barrier organs start expressing receptors, allowing the detection of a broad spectrum of pathogens, especially fungi and bacteria. We additionally identified a decrease in the inhibitory CTLRs *Clec4a2*, *Clec12a*, and particularly *Clec1b* (also known as CLEC-2) (45, 46). It is possible that the loss of inhibitory CTLRs may be the contributing factor towards the inflammatory response seen with antibody transfer. For example, Clec1b deficiency has been linked to systemic edema (46). Further mechanistic studies would provide insight into the role of inhibitory CTLRs.

In summary, we demonstrate the transformation of the neutrophil transcriptome from blood to blister in a well-characterized model of EBA. While we have demonstrated that Dectin-2, Dectin-3, and Mincle are most notable on skin-infiltrating neutrophils, our knockout models suggest that they do not individually contribute to the pathogenic role of neutrophils in EBA.

## Data availability statement

The datasets presented in this study can be found in online repositories. The names of the repository/repositories and accession number(s) can be found below: https://www.ncbi.nlm.nih.gov/geo/query/GSE231971.

## Ethics statement

The studies involving humans were approved by Rush University Medical Center. The studies were conducted in accordance with the local legislation and institutional requirements. The participants provided their written informed consent to participate in this study. The animal study was approved by Rush University Medical Center (IACUC No 20-079) or the state government of Schleswig-Holstein. The study was conducted in accordance with the local legislation and institutional requirements.

## Author contributions

CG-J: Formal Analysis, Methodology, Visualization, Writing – original draft, Writing – review & editing, Data curation. PS: Investigation, Visualization, Writing – review & editing. JL: Investigation, Writing – review & editing. MZ: Investigation, Methodology, Writing – review & editing. LB: Investigation, Writing – review & editing. PP: Investigation, Writing – review & editing. JG-T: Investigation, Validation, Writing – review & editing. SM: Funding acquisition, Investigation, Writing – review & editing. CC: Investigation, Writing – review & editing. MS: Investigation, Methodology, Writing – review & editing. MF: Resources, Writing – review & editing. TH: Resources, Writing – review & editing. NI: Resources, Writing – review & editing. TR: Investigation, Resources, Writing – review & editing. KB: Resources, Writing – review & editing. RL: Resources, Writing – review & editing. CS: Writing – original draft, Writing – review & editing. KA: Conceptualization, Formal Analysis, Methodology, Project administration, Resources, Supervision, Validation, Visualization, Writing – original draft, Writing – review & editing.

## Supplementary material

Supplementary Figure 1 Quality control metrics of scRNA-seq data from blood and ear skin. (Left) Probability density scores showing putative doublets/multiplets demarcated at the bimodal distribution of cells’ doublet score. (Middle) Doublets/multiplets were projected on two-dimensional UMAPs and colored black. Singlets were then processed for quality control filtering. Singlets with abnormal gene numbers (350 > genes/cell < 5,000), high mitochondrial gene percentage (<10%), and outliers were removed. Viable singlets were used for downstream query and comparative analyses. (Right) Violin plots showing distribution of features and counts expression levels, and mitochondrial gene percentage in viable singlets used for downstream analyses. EBA, epidermolysis bullosa acquisita; IgG, immunoglobulin G; UMAP, Uniform Manifold Approximation and Projection; WBC, whole blood cells; QC, quality control.

Supplementary Figure 2 Immune populations in whole blood. **(A)** Relative percentage of immune cells in EBA (red) and control treatment (yellow), demonstrate a relative increase in immune cells in neutrophils, mast, and myeloid cells in EBA. Percentage of immune cells in EBA **(B)** and control-IgG injections **(C)** demonstrates shift from lymphocytes to myeloid cells in EBA. EBA, epidermolysis bullosa acquisita; IgG, immunoglobulin G; WB, whole blood; NKC, natural killer cell; Treg, regulatory T cell.

Supplementary Figure 3 Expression of CLEC genes on gated neutrophils in experimental EBA blood neutrophils. Two-dimensional feature plots showing expression of CLEC genes, including Clec4d, Clec4e, Clec4n, Clec4a2, Clec12a, and Clec1b in EBA skin versus EBA blood versus IgG control ear, blood, and skin neutrophils. Data are shown as a function of total bioinformatically gated, S100a8+, S100a9+, and Ly6g+ neutrophil cells on skin (left) and of gated neutrophils (right). Gray Light green, low normalized gene expression based on normalized counts; black, high normalized gene expression based on normalized counts. EBA, epidermolysis bullosa acquisita; IgG, immunoglobulin G; UMAP, Uniform Manifold Approximation and Projection; Lo, low; Hi, high; Norm, Cts, normalized counts.

Supplementary Table 1 Differentially expressed genes between EBA and control blood neutrophils.

Supplementary Table 2 Differentially expressed genes between lesional and blood neutrophils in experimental EBA.

Supplementary Table 3 Summary alignment metrics for each group.

Supplementary Table 4 List of significant gene ontology findings for neutrophils.
