# Supplementary figures and images for: C-type lectin receptor expression is a hallmark of neutrophils infiltrating the skin in epidermolysis bullosa acquisita
Source: Front Immunol. 2023 Sep 20;14:1266359. doi: 10.3389/fimmu.2023.1266359 (PMC10548123; doi:10.3389/fimmu.2023.1266359)

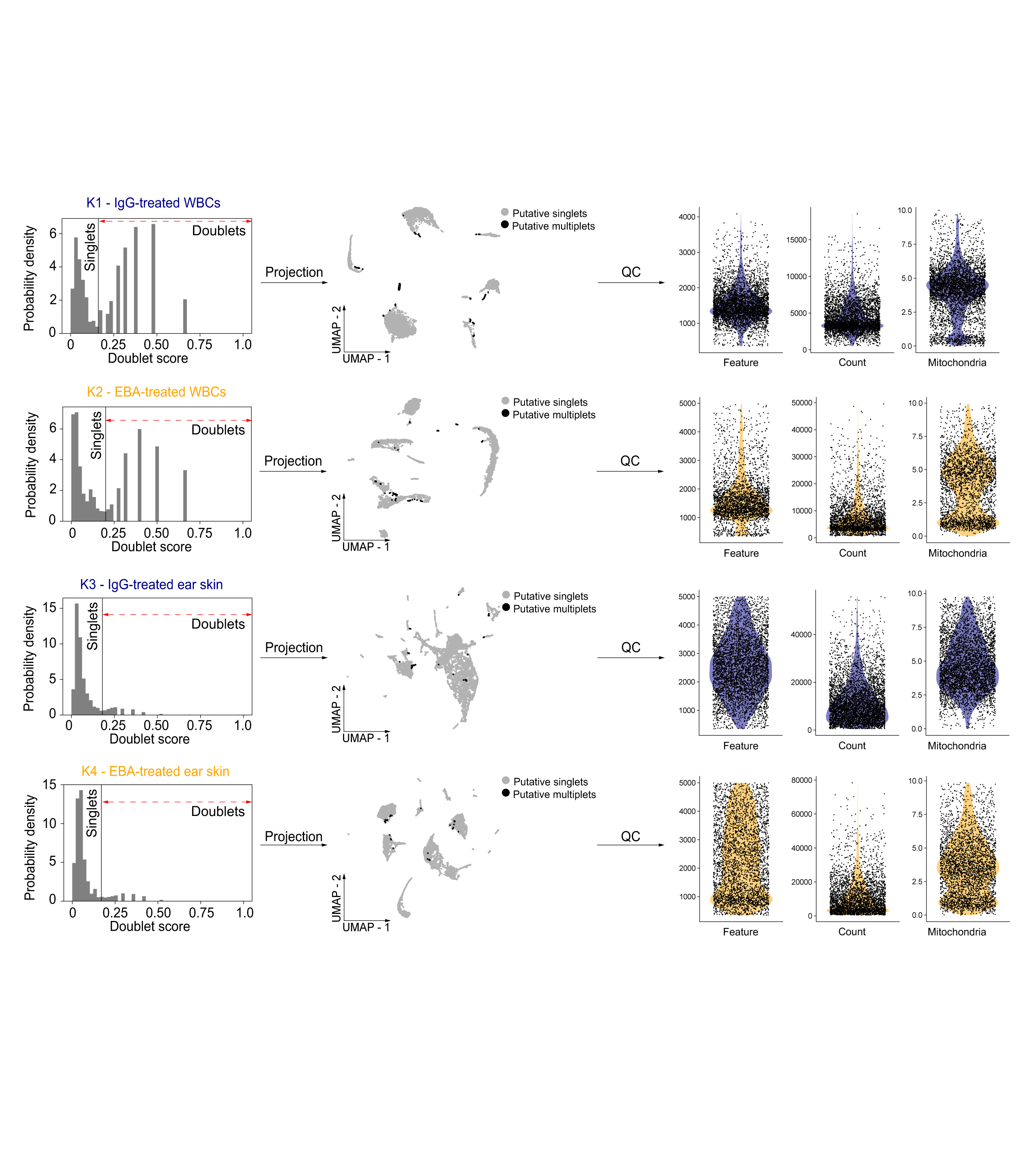

Supplement: Supplementary Figure 1 — Quality control metrics of scRNA-seq data from blood and ear skin. (Left) Probability density scores showing putative doublets/multiplets demarcated at the bimodal distribution of cells’ doublet score. (Middle) Doublets/multiplets were projected on two-dimensional UMAPs and colored black. Singlets were then processed for quality control filtering. Singlets with abnormal gene numbers (350 > genes/cell < 5,000), high mitochondrial gene percentage (<10%), and outliers were removed. Viable singlets were used for downstream query and comparative analyses. (Right) Violin plots showing distribution of features and counts expression levels, and mitochondrial gene percentage in viable singlets used for downstream analyses. EBA, epidermolysis bullosa acquisita; IgG, immunoglobulin G; UMAP, Uniform Manifold Approximation and Projection; WBC, whole blood cells; QC, quality control. [file Image_1.jpeg]

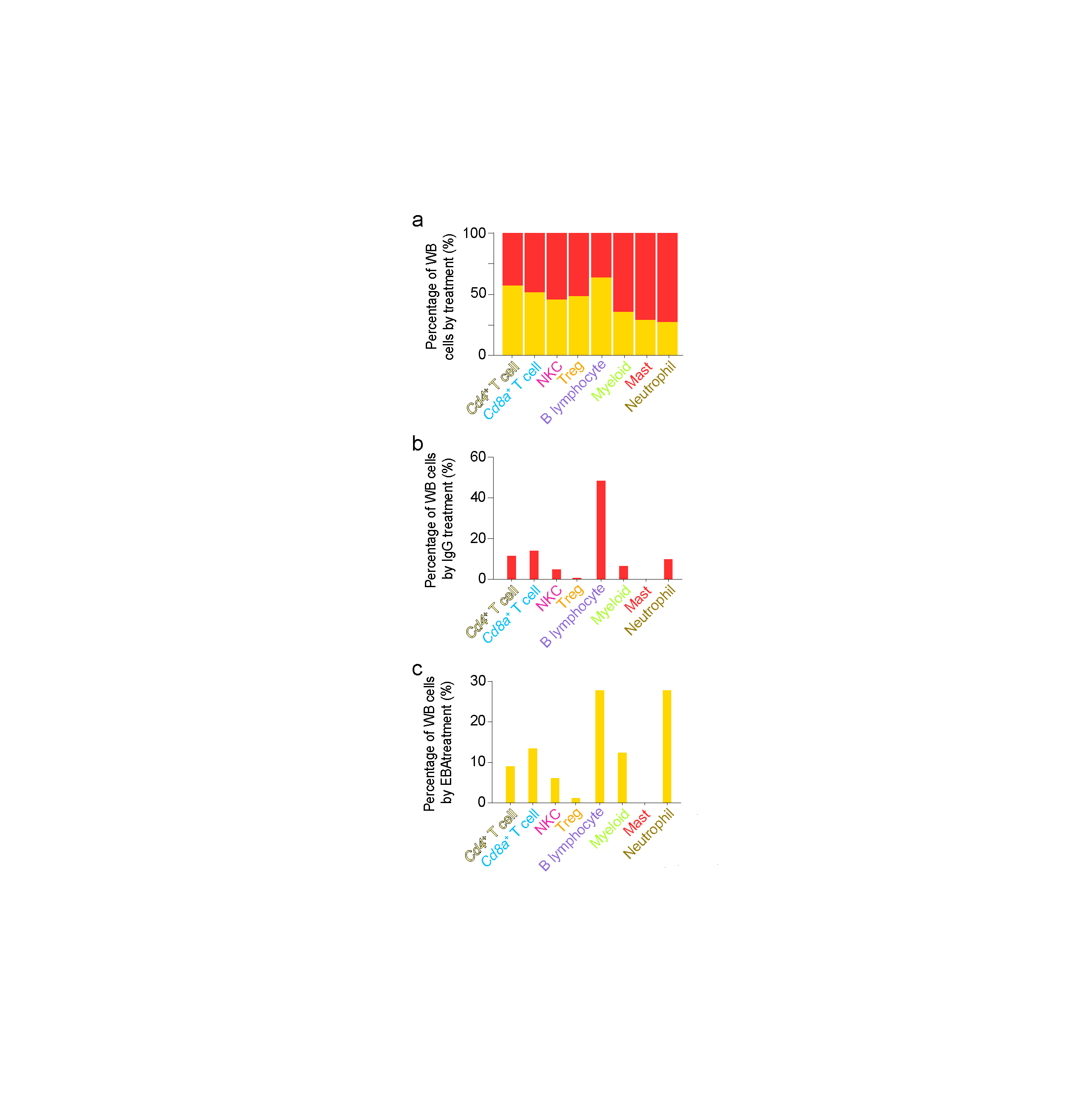

Supplement: Supplementary Figure 2 — Immune populations in whole blood. (A) Relative percentage of immune cells in EBA (red) and control treatment (yellow), demonstrate a relative increase in immune cells in neutrophils, mast, and myeloid cells in EBA. Percentage of immune cells in EBA (B) and control-IgG injections (C) demonstrates shift from lymphocytes to myeloid cells in EBA. EBA, epidermolysis bullosa acquisita; IgG, immunoglobulin G; WB, whole blood; NKC, natural killer cell; Treg, regulatory T cell. [file Image_2.jpeg]

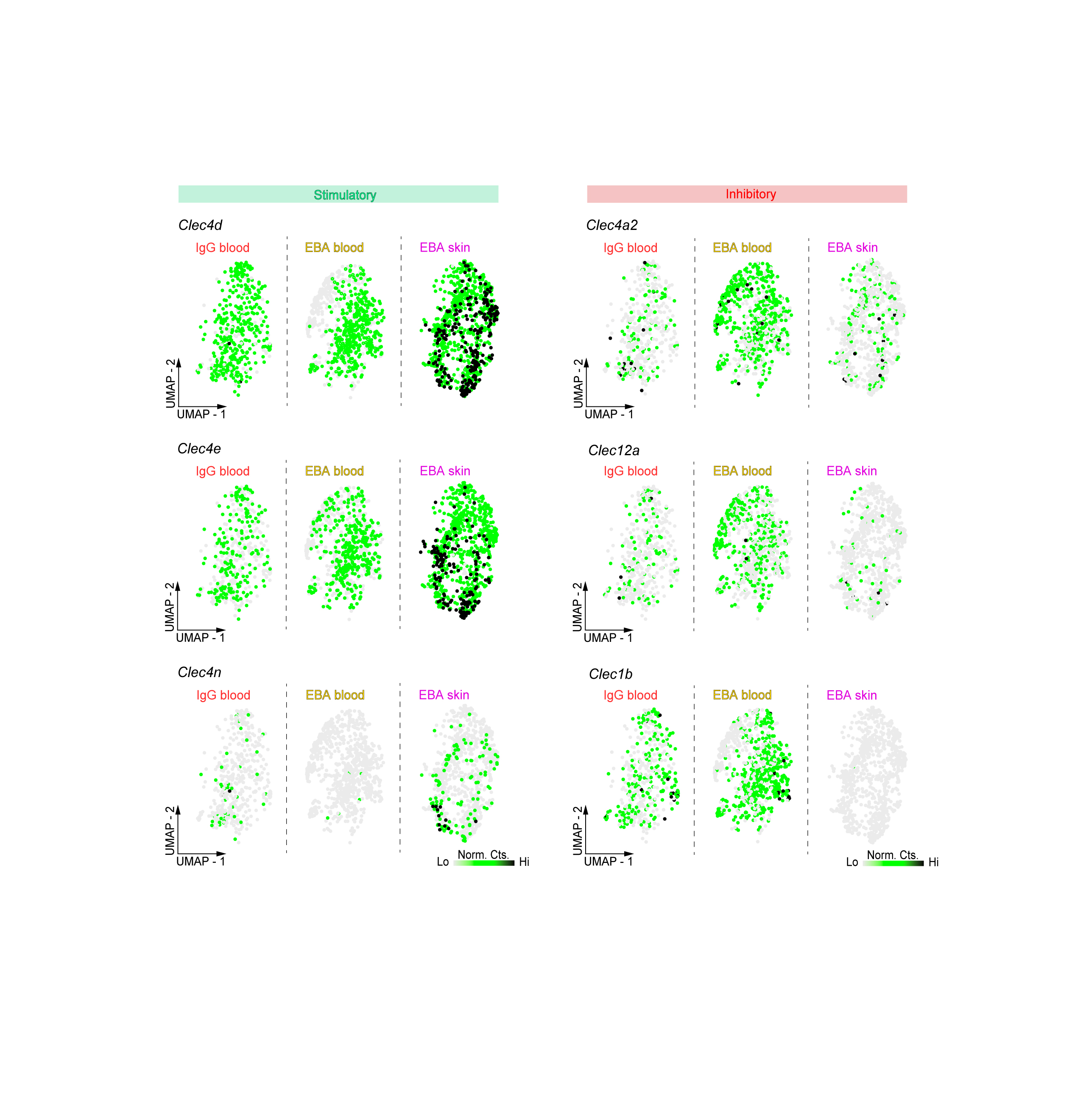

Supplement: Supplementary Figure 3 — Expression of CLEC genes on gated neutrophils in experimental EBA blood neutrophils. Two-dimensional feature plots showing expression of CLEC genes, including Clec4d, Clec4e, Clec4n, Clec4a2, Clec12a, and Clec1b in EBA skin versus EBA blood versus IgG control ear, blood, and skin neutrophils. Data are shown as a function of total bioinformatically gated, S100a8+, S100a9+, and Ly6g+ neutrophil cells on skin (left) and of gated neutrophils (right). Gray Light green, low normalized gene expression based on normalized counts; black, high normalized gene expression based on normalized counts. EBA, epidermolysis bullosa acquisita; IgG, immunoglobulin G; UMAP, Uniform Manifold Approximation and Projection; Lo, low; Hi, high; Norm, Cts, normalized counts. [file Image_3.jpeg]
